# Controllable and Scale-Up Synthesis of Nickel-Cobalt Boride@Borate/RGO Nanoflakes *via* Reactive Impingement Mixing: A High-Performance Supercapacitor Electrode and Electrocatalyst

**DOI:** 10.3389/fchem.2022.874675

**Published:** 2022-04-12

**Authors:** Yudan Qian, Yechao Wu, Fan Gu, Zhiming Zhou, Zaimei Huang, Xinyue Tang, Shuang Pan, Shangcong Zhang, Shinan Chen, Qingcheng Zhang, Yihuang Chen, Shun Wang

**Affiliations:** ^1^ College of Chemistry and Materials Engineering, Wenzhou University, Wenzhou, China; ^2^ Low Voltage Apparatus Technology Research Center of Zhejiang, Wenzhou University, Wenzhou, China; ^3^ Technology Institute of Wenzhou University in Yueqing, Wenzhou, China; ^4^ Zhejiang Zheneng Wenzhou Electric Power Generation Co., Ltd., Wenzhou, China

**Keywords:** reactive impingement mixing, nickel-cobalt boride@borate/RGO, supercapacitor, electrocatalyst, scale-up synthesis

## Abstract

Large-scale synthesis of graphene-based nanomaterials in stirred tank reactor (STR) often results in serious agglomeration because of the poor control during micromixing process. In this work, reactive impingement mixing is conducted in a two-stage impinging jet microreactor (TS-IJMR) for the controllable and scale-up synthesis of nickel-cobalt boride@borate core-shell nanostructures on RGO flakes (NCBO/RGO). Benefiting from the good process control and improved micromixing efficiency of TS-IJMR, NCBO/RGO nanosheet provides a large BET surface area, abundant of suitable mesopores (2–5 nm), fast ion diffusion, and facile electron transfer within the whole electrode. Therefore, NCBO/RGO electrode exhibits a high specific capacitance of 2383 F g^−1^ at 1 A g^−1^, and still retains 1650 F g^−1^ when the current density is increased to 20 A g^−1^, much higher than those of nickel boride@borate/RGO (NBO/RGO) and cobalt boride@borate/RGO (CBO/RGO) synthesized in TS-IJMR, as well as NCBO/RGO-S synthesized in STR. In addition, an asymmetric supercapacitor (NCBO/RGO//AC) is constructed with NCBO/RGO and activated carbon (AC), which displays a high energy density of 53.3 W h kg^−1^ and long cyclic lifespan with 91.8% capacitance retention after 5000 charge-discharge cycles. Finally, NCBO/RGO is used as OER electrocatalyst to possess a low overpotential of 309 mV at a current density of 10 mA cm^−2^ and delivers a good long-term durability for 10 h. This study opens up the potential of controllable and scale-up synthesis of NCBO/RGO nanosheets for high-performance supercapacitor electrode materials and OER catalysts.

## 1 Introduction

With the continuous consumption of fossil resources and deterioration of the global environment, the development of efficient energy storage and conversion systems is urgent for utilizing green, renewable but intermittent energy sources, such as hydrogen energy, tidal energy, solar power etc. Among various energy storage devices, supercapacitors have received enormous attention owing to their outstanding advantages of fast charge–discharge capability, high power density, long cycling lifespan, and good operational safety (Liu et al., 2020; Zhao et al., 2021b; Pan et al., 2022). The electrode materials of commercial supercapacitors are mainly porous carbonaceous materials (Zhang et al., 2019a; Jin et al., 2019; He et al., 2020), whose energy storage mechanisms are based on the principle of electrochemical double layer capacitance (EDLC). Although these carbonaceous materials have high electrical conductivity and good cycling stability, the low energy densities hamper their further application in large energy storage systems (Jin et al., 2018). In addition, hydrogen production from electrochemical water splitting can effectively solve the current energy crisis and environmental problems. However, the oxygen evolution reaction (OER) is the major bottleneck due to its high reaction overpotential and sluggish reaction kinetics, thus severely reducing the overall efficiency of hydrogen generation (Teng et al., 2020; Zhang et al., 2021). Although ruthenium- and iridium-based electrocatalysts have excellent OER efficiency, the high cost and low natural abundance of precious metals seriously impede their widespread applications (Laha et al., 2019; She et al., 2022). Therefore, it is imperative for us to develop efficient and cost-effective bifunctional electrode materials for advanced energy storage and conversion devices.

In the past decade, transition metal borides (TMBs) have been particularly attractive for applications in hydrogen evolution reaction, OER, lithium-ion batteries, and supercapacitors because of their abundant electroactive sites arising from the electron deficiency of boron atoms (Park et al., 2017; Dong et al., 2018; Chen et al., 2019b; Guo et al., 2019). Among them, Ni-B is extensively studied as a pseudocapacitive electrode material and an OER electrocatalyst due to its large theoretical specific capacitance and superior intrinsic activity, respectively (Chen et al., 2019a; Li et al., 2019). However, Ni-B has a poor electrical conductivity and is prone to structural collapse during long-term stability test (Li et al., 2017). Co-B possesses a high electrical conductivity in the order of 10^3^ S cm^−1^ (that is, similar to metallic Co) (Wang et al., 2017). Therefore, the coordination of Ni/Co species to form bimetallic boride potentially tailors the electrical structure to address both the electrical conductivity and electrochemical stability (Xu et al., 2017). Meanwhile, constructing Ni-B with graphene is also an effective strategy to boost the electrochemical activity of Ni-B, since graphene sheet not only constructs a two-dimensional (2D) conductive network for Ni-B to promote charge transfer and accelerate the electrolyte ions transport within the whole electrode, but also prevents the structural deformation and volume expansion of Ni-B particles during long-term stability test (Chen et al., 2019b). In addition, it should be concerned that surface metal borides are easily oxidized since oxygen is more electro-negative than boron, therefore, the aforementioned TMBs are actually transition metal borides@borates (TMBOs) (Masa et al., 2016; Masa et al., 2017; Tan et al., 2019).

TMBOs are usually generated by the co-precipitation of metal ions with reducer (e.g., NaBH_4_). It is generally suggested that a homogeneous supersaturation, which is generated through delicate mixing of two reactant solutions, is essential for the precipitation of nanomaterials with good product quality because of the highly nonlinear dependency of nucleation rate on supersaturation level (Bałdyga et al., 2005; Chen et al., 1996; Lafficher et al., 2018). Large-scale synthesis of nanomaterials is usually performed in stirred tank reactors (STRs). However, particle nucleation is always instantaneous with characteristic timescale at the same order of magnitude or smaller than the timescale of the mixing process (Kaluza and Muhler, 2009), hence fast precipitation may have already occurred or completed before the reactant solutions accomplish a homogeneous supersaturation in STR due to its poor mixing and mass transfer efficiency (Lince et al., 2009). STRs are also difficult to precisely control experimental parameters, e.g., reactant concentration and pH values. Even under the so-called “constant” pH precipitation at a slow feeding rate, the local concentration, pH, and supersaturation in STR vary temporally and spatially when a drop of reactant solution is titrated into STR, where the previously formed precipitates and these dissolved ions has already coexisted (Bhattacharya and Kresta, 2004). Therefore, particle nucleation, growth, and phase transformation may take place simultaneously in STR, which makes it difficult to precisely maintain the product quality of nanomaterials (e.g., morphology, purity and size distribution) (Cheng et al., 2009; Alexandrov, 2014). In addition, large-scale synthesis of graphene-based composites in STR often result in serious self-agglomeration owing to the poor control during the mixing process, and the inevitable coagulation of negatively charged GO flakes premixes with the positively charged metal ions (Li et al., 2008; Ma et al., 2015). As a consequence, TMBO/graphene generated in STR may differ significantly in the morphologies, microstructures, composition distributions, and electrochemical performances. Thus, it is of vital importance to develop a more efficient and controllable technique for the scale-up synthesis of TMBO/graphene composites. The core technology lies in the enhanced micromixing of fluids through chemical process intensification technology to put the reaction system in a more homogeneous nucleation environment (Schwarzer and Peukert, 2004; Li et al., 2012).

In the past decades, researchers have developed a series of novel reactors such as microchannel reactors, capillary microreactors, and static mixers to enhance the micromixing of fluids for the controllable synthesis of nanomaterials (Meincke et al., 2017; Rao et al., 2019; Yang et al., 2019). However, these microreactors require precision machining and are prone to clogging during precipitation, so the yield of synthetic nanomaterials is very limited. In addition, these microreactors are usually suitable for only two fluid feeds, hence a premixing of the metal salt and graphene oxide (GO) solution in STR is required before the mixture solution is injected into the microreactor to precipitate with reducer for the controllable synthesis of TMBO/RGO composite. However, when negatively charged GO sheets are premixed with positively charged metal ions in STR, not only induces serious coagulation of GO sheets but also introduces extra labor-intensive and time-consuming operations (Li et al., 2008). Therefore, it is urgent for us to develop a micromixing-intensified platform that integrates the homegeneous premixing and subsequent reactive particle precipitation in a single streamlined process.

Herein, a two-stage impinging jet microreactor (TS-IJMR) with homogeneous premixing and precipitation functions is designed for the controllable and scale-up synthesis of nickel-cobalt boride@borate/RGO (NCBO/RGO) composites. TS-IJMR is composed of two micro T-junctions and several capillary cubes. Firstly, Ni^2+^/Co^2+^ and GO solutions are impinged rapidly within the first micro T-junction to get homogeneously premixed. Immediately (after ∼0.1 s), the mixed Ni^2+^/Co^2+^/GO solutions flows into the second micro T-junction and reversely collides with the reducer at high velocities, thus creating a high energy dissipation zone for the intensified micromixing and consequently the uniform supersaturation level (Guo et al., 2017). In addition, high-speed impingement of the three fluids in TS-IJMR enables yields >200 ml min^−1^, thus significantly improving productivity and reproducibility. Therefore, the as-prepared NCBO/RGO-3 displays a large specific capacitance of 2383 F g^−1^ at 1 A g^−1^, good rate capability and excellent cycling stability. An asymmetric supercapacitor (NCBO/RGO//AC) assembled with NCBO/RGO-3 and activated carbon (AC) has exhibited a high energy density of 53.3 W h kg^−1^, coupling with 91.8% of capacitance retention after 5000 fast charge/discharge cycles. In addition, NCBO/RGO-3 displays a low overpotential of 309 mV at 10 mA cm^−2^ for OER.

## 2 Experimental Section

### 2.1 Construction of Two-Stage Impinging Jet Microreactor

TS-IJMR consists of three constant-flow pumps (2 PB-20005II, Beijing Xingda Science and Technology Development Co. Ltd.), two micro T-junctions (SS-1UTF, Beijing Xiongchuan Technology Co. Ltd.), and several stainless-steel capillary tubes (inner diameter *d*_i_ = 0.6 mm). Additionally, three capillary tubes link to the first micro T-junction to construct an internal structure similar to a “T-shaped micromixer” for the homogeneous mixing of Ni^2+^/Co^2+^ and GO solutions. At the second micro T-junction, two capillary tubes are aligned coaxially and inversely. However, the second micro T-junction outlet is no longer connected to the capillary tube but forms an enlarged channel, which can effectively reduce the blockage problem during precipitation. After the uniformly mixed Ni^2+^/Co^2+^/GO solution collides with reducer within the second micro T-junction at high velocities, the generated precipitates will immediately downward into other vessels for aging. Precipitation and aging processes are hence separated in two containers in the TS-IJMR route, which can provide a more uniform and steady environment for both particle nucleation and crystal growth on top of the enhanced micromixing performance and better process control of TS-IJMR. The geometric structures of TS-IJMR are depicted in Supplementary Figure S1.

### 2.2 Synthesis of Nickel-Cobalt Boride@Borate/RGO Composites by Two-Stage Impinging Jet Microreactor (i.e., NCBO-RGO)

Nickel sulfate hexahydrate (NiSO_4_ · 6H_2_O), Cobalt nitrate hexahydrate (Co(NO_3_)_2_ · 6H_2_O) and potassium hydroxide (KOH) were provided by Aladdin Reagent (Shanghai) Co., Ltd. Potassium borohydride (KBH_4_) was purchased from Shanghai Macklin Biochemical Co., Ltd. All the chemicals were analytical grade and used without further purification.

**FIGURE 1:**
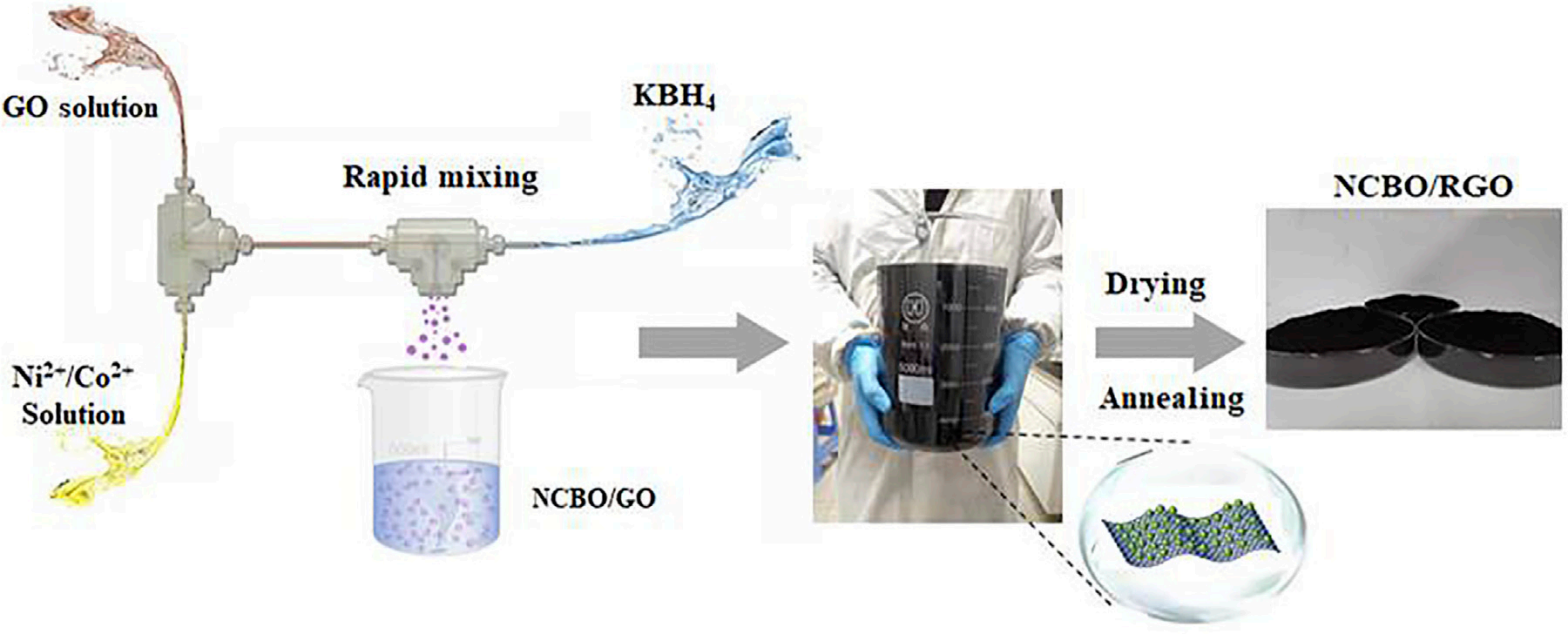
Schematic illustration of the scale-up synthesis of NCBO/RGO in TS-IJMR.

The controllable and scale-up synthesis of NCBO/RGO composites in TS-IJMR is shown in Figure 1. Firstly, 0.1 mol L^−1^ NiSO_4_ and Co(NO_3_)_2_ mixed solution (Ni^2+^: Co^2+^ = 3:1) and 1 mg ml^−1^ GO solution were pumped into the first micro T-junction at constant volumetric flow rates to obtain a uniformly mixed Ni^2+^/Co^2+^/GO solution. Then, the mixture immediately flowed into the second micro T-junction to produce NCBO/GO-3 (i.e., Ni^2+^/Co^2+^ molar ratios of 3:1) precursor by reversely high-speed impact with 0.1 mol L^−1^ KBH_4_ solution. The volumetric flow rates of Ni^2+^/Co^2+^ solution, GO solution, and KBH_4_ solution were maintained at 80, 80, and 160 ml min^−1^, respectively, with the corresponding flow velocities of 4.76, 4.76, and 9.52 m s^−1^, respectively. The precipitate generated from the second micro T-junction immediately flowed into a storage tank and aged for 6 h under room temperature with vigorous agitation. Next, the NCBO/GO-3 precursor was washed 3 times each by deionized water and anhydrous ethanol before being dried overnight at 80°C. Finally, the NCBO/GO-3 precursor was calcined at 300°C in an argon atmosphere for 3 h. The GO was successfully converted to reduced graphene oxide (RGO), thus producing NCBO/RGO-3. In addition, the samples synthesized in the same TS-IJMR route with Ni^2+^/Co^2+^ molar ratios of 1:2, 1:1, 2:1, and 4:1 were denoted as NCBO/RGO-0.5, NCBO/RGO-1, NCBO/RGO-2 and NCBO/RGO-4, respectively.

With the same synthesis method, nickel boride@borate/RGO (NBO/RGO) and nickel boride@borate/RGO (CBO/RGO) were synthesized by replacing the Ni-Co mixed solution with 0.1 mol ml^−1^ NiSO_4_ and 0.1 mol ml^−1^ Co(NO_3_)_2_ solution, respectively.

### 2.3 Synthesis of Nickel-Cobalt Boride@Borate/RGO by Stirred Tank Reactor (i.e., NCBO-RGO-S)

The NCBO/RGO-S was synthesized by parallel precipitation in STR. First, 0.1 mol L^−1^ NiSO_4_/Co(NO_3_)_2_ mixture solution (Ni^2+^: Co^2+^ = 3:1) and 1 mg ml^−1^ GO solution were added dropwisely at 10 ml min^−1^ to a beaker with vigorous stirring to obtain the mixed Ni^2+^/Co^2+^/GO solution (Supplementary Figure S1). Then, Ni^2+^/Co^2+^/GO mixture solution and 0.1 mol L^−1^ KBH_4_ solution were added dropwise at 10 ml min^−1^ to a beaker with vigorous stirring. After titration, the suspension was further aged for 6 h under room temperature with vigorous agitation. The subsequent steps are consistent with those of TS-IJMR. The resulting sample was labeled “NCBO/RGO-S”.

### 2.4 Characterizations

The crystal structure and phase composition of the TMBO/RGO samples were characterized using a X-ray diffraction analyzer (XRD, Bruker D8) with Cu K*α* radiation (*λ* = 0.15406 nm) and X-ray photoelectron spectra (XPS, ESCALAB 250) with an Al Kα radiation source. Fourier transform infrared spectrum (FT-IR) was conducted in the wavelength of 4000–400 cm^−1^ with a Bruker Vetex 70 spectrometer. The surface morphologies of the TMBO/RGO samples were observed with scanning electron microscopy (SEM, Nova NanoSEM 200) and transmission electron microscopy (TEM, JEOL JEM-2100). High-resolution transmission electron microscopy (HR-TEM), energy-dispersive X-ray spectrometer (EDS) and selected area electron diffraction (SAED) were performed on the same transmission electron microscopy. The nitrogen adsorption–desorption isotherms of the TMBO/RGO samples were recorded by a Micromeritics analyzer (ASAP 2020M), while their corresponding specific surface areas were determined from the Brunauer–Emmett–Teller (BET) equation.

### 2.5 Electrochemical Measurement

#### 2.5.1 Supercapacitor

The electrochemical properties of all synthetic samples were evaluated in a three-electrode configuration using an electrochemical workstation (CHI760E, Shanghai Chenhua Co., Ltd., China). The working electrode was made by mixing 80 wt% of TMBO/RGO sample, 15 wt% of acetylene black, and 5 wt% of polytetrafluoroethylene (PTFE) in N-methyl pyrrolidone (NMP) to be fully ground into a slurry and then evenly coated on 1 × 1 cm^2^ nickel foam before being dried at 60°C. The average mass loading of the active material on nickel foam was about 3 mg. The Hg/HgO and platinum electrodes were chosen as the reference and counter electrodes, respectively, with 2 M KOH as the electrolyte. The specific capacitances *C*_sp_ (F g^−1^) of the TMBO/RGO samples were determined from Eq. 1.

$$C_{\text{sp}}=\frac{I\times \text{Δ}t}{m\times \text{Δ}U}$$ (1)

Where *I* (A) is the charge/discharge current, ∆*t* is the discharge time, *m* (g) is the mass loading of the active material, and ∆*U* is the operating potential window.

The asymmetric supercapacitor NCBO/RGO//AC was assembled with NCBO/RGO-3 as the positive electrode material and AC as the negative electrode material. They are separated by a cellulose acetate membrane (TF4030, NKK, Japan). According to the following charge conservation equation, the mass ratio between the positive and negative materials (m^+^/m^−^) was finally determined to be 0.35.

$$\frac{m^{+}}{m^{-}}=\frac{C_{sp}^{-}\times \text{Δ}U^{-}}{C_{sp}^{+}\times \text{Δ}U^{+}}$$ (2)

The energy density (*E*, W h kg^−1^) and the power density (*P*, W kg^−1^) of NCBO/RGO//AC were calculated by the following equations, respectively:

$$E=\frac{C_{\text{cell}}\times \text{Δ}U^{2}}{2\times 3.6}$$ (3)

$$P=\frac{E\times 3600}{\text{Δ}t}$$ (4)

Where *C*_cell_ is the specific capacitance of NCBO/RGO//AC calculated based on the total mass of positive and negative active materials, and Δ*U* (V) is the voltage window of the asymmetric supercapacitor.

#### 2.5.2 Oxygen Evolution Reaction Test

The OER performance of different catalysts was tested using CHI760E in a three-electrode system. The catalyst was made by dispersing 6 mg of NCBO/RGO-3 powder, 3 mg of Ketjen black in 50 ml of Nafion solution (5 wt%) and 1 ml of ethanol, and then sonicated for 1 h to obtain a homogeneous slurry. Next, 20 μL of catalyst slurry was uniformly dispersed on a 5 mm glassy carbon electrode (loading of 0.6 mg cm^−2^) as the working electrode. The carbon rod and Hg/HgO electrode were acted as the counter and reference electrodes, respectively. An OER performance test was performed in 1 M KOH solution by linear sweep voltammetry (LSV). Additionally, iR correction was performed for all polarization curves to account for any uncompensated resistance, and *E*_RHE_ = *E*_Hg/HgO_ + 0.098 + 0.0591 pH-*iR*.

For comparison of the performance of catalysts, 20 wt% RuO_2_ catalysts were also studied. The same methods and loadings were used for preparation and testing.

## 3 Results and Discussion

### 3.1 Morphological and Structural Characterizations of Transition Metal Borides@Borates/RGO Samples

**FIGURE 2:**
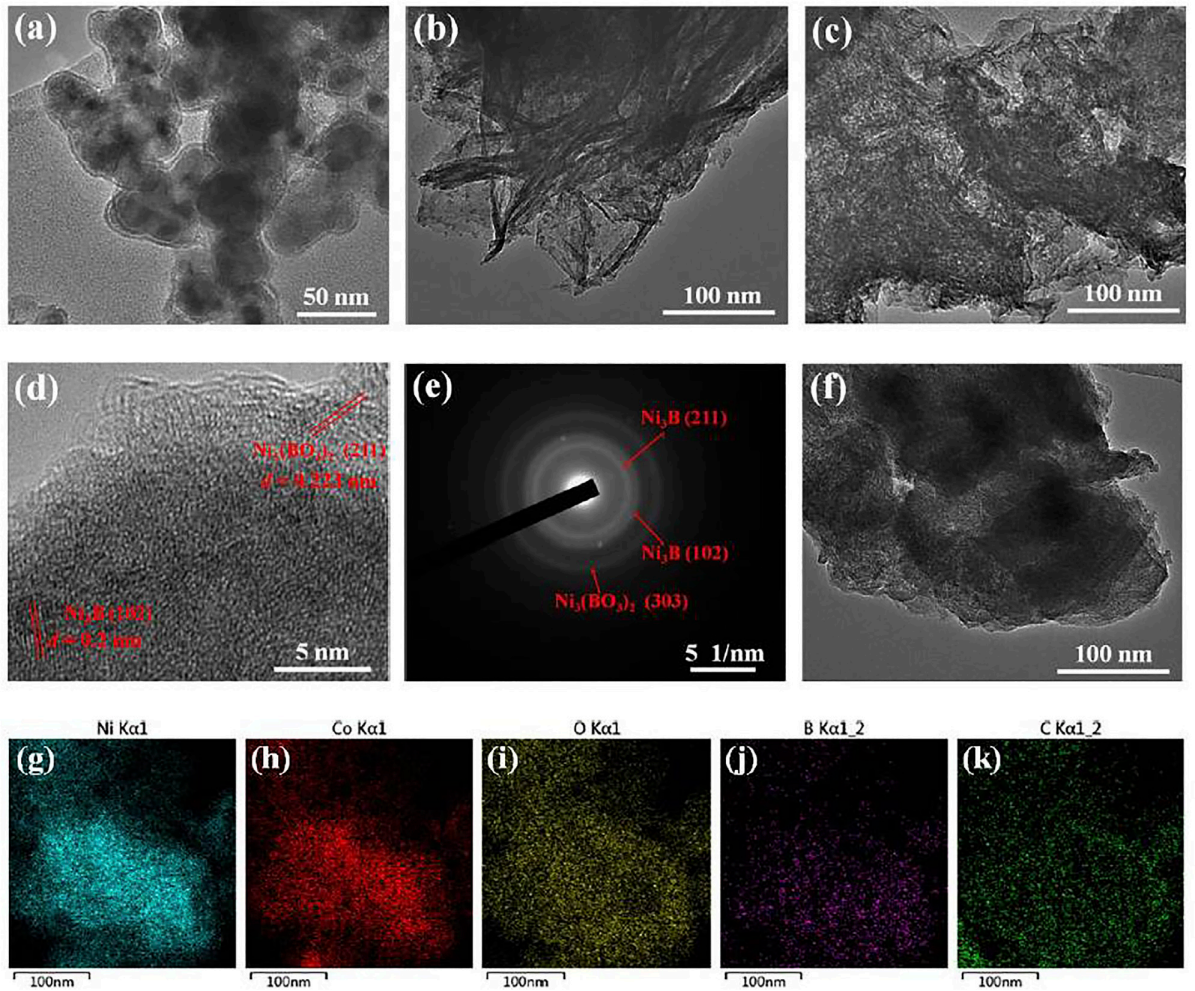
**(A–C)** TEM images of NBO/RGO, CBO/RGO and NCBO/RGO-3, respectively; **(D)** HR-TEM image of NCBO/RGO-3; **(E)** SAED image of NCBO/RGO-3; **(F)** TEM image of NCBO/RGO-S that synthesized in STR; **(G–I)** EDS mapping of different elements in NCBO/RGO-3.

The morphologies of TMBO/RGO samples were observed with electron microscopy. As shown by SEM images in Supplementary Figure S3, all the three TMBO/RGO samples consist of TMBO nanoparticles that tightly wrapped with RGO flakes. TEM images of the three TMBO/RGO samples are shown in Figures 2A–C. With respect to NBO/RGO, core-shell spherical NCO particles with a particle size of 30–40 nm and a shell layer thickness of 2–3 nm are loaded on RGO flakes (Figure 2A), while CBO/RGO contains many long strips with lengths of several hundred nanometers that are randomly dispersed on RGO flakes (Figure 2B). In addition, it can be seen from Figure 2C that the NCBO/RGO-3 possesses nanoflake structure without obvious agglomeration and accumulation. The corresponding HR-TEM image shows faint lattice fringes of 0.223 nm and interplanar spacing of 0.2 nm, which can be attributed to the (211) crystal plane of Ni_3_(BO_3_)_2_ and the (102) crystal planes of Ni_3_B, respectively. The low-resolution lattice fringes suggest that NCBO/RGO-3 has a low crystallinity (Figure 2D). The presence of (303) crystal plane of Ni_3_(BO_3_)_2_, (102) and (211) crystal planes of Ni_3_B in NCBO/RGO-3 is further confirmed by the SAED in Figure 2E. In addition, Figure 2F reveals the presence of agglomerated lamellar structure in the NCBO/RGO-S synthesized by STR route. Finally, the EDS mapping demonstrates a uniform distribution of Ni, Co, O, B, and C elements within the NCBO/RGO-3 (Figure 2G–K).

**FIGURE 3:**
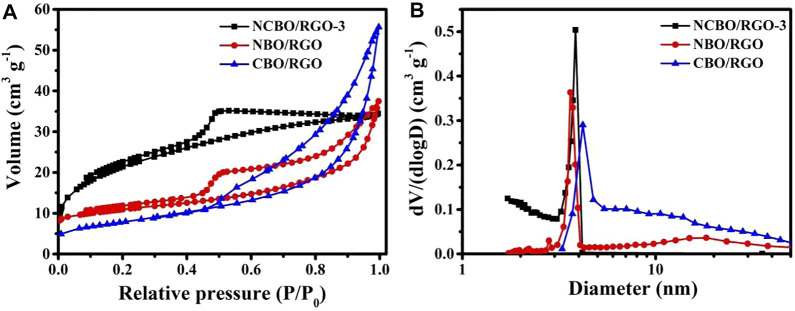
**(A)** N_2_ adsorption/desorption isotherms; **(B)** Pore size distributions of the three TMBO/RGO samples.

The N_2_ adsorption-desorption isotherms are shown in Figure 3A to study the textural properties and pore structures of the three TMBO/RGO samples. All samples show the typical IV adsorption behaviors with distinct hysteresis loops in the relative pressure (P/P_0_) range of 0.4–1.0, indicating they mainly comprises mesoporous structures. The pore size distributions derived from their corresponding desorption curves are shown in Figure 3B. All the samples display a sharp and narrow peak at 2–5 nm that presumably stem from the internal pore sizes of each TMBO/RGO. In addition, the pore size distribution peaks of 10–50 nm existed for NBO/RGO and CBO/RGO may be originated from the interstices between the loaded particles on the RGO sheet. These abundant pores, especially the 2–5 nm mesopores, can increase the specific surface area of the material (Borchardt et al., 2018). Therefore, the BET specific surface area of NCBO/RGO-3 is as high as 62.37 m^2^/g, which is much larger than 30.31 and 42.89 m^2^/g of NBO/RGO and CBO/RGO. A larger specific surface area provides more superficial electroactive sites to participate in redox reactions, while abundant pores can shorten the transport channels from electrolyte ions to the internal pores of electrode materials (Borchardt et al., 2018). Therefore, NCBO/RGO-3 with relatively large BET specific surface area and abundant pore channels is expected to display high electrochemical performance.

**FIGURE 4:**
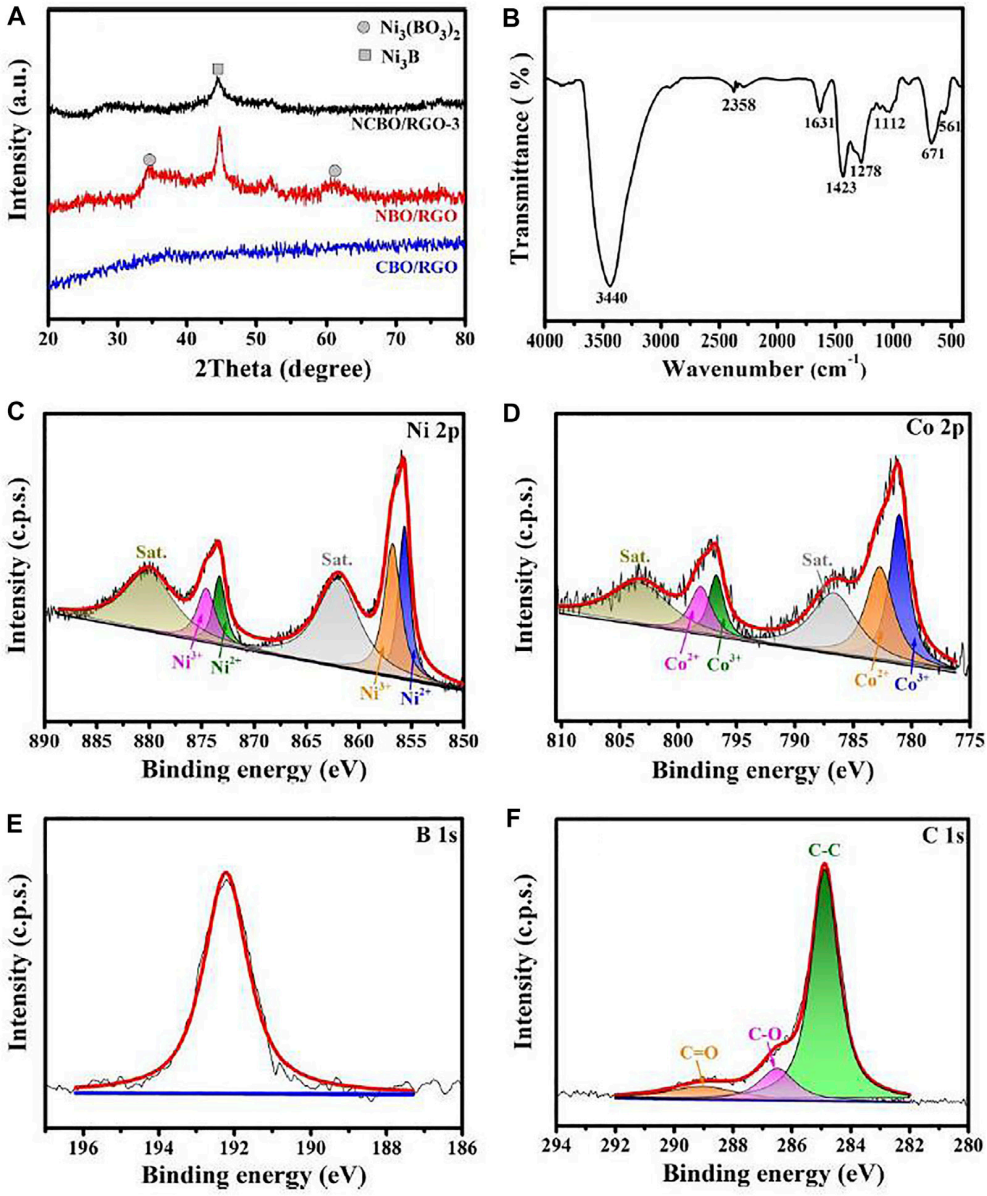
**(A)** XRD patterns of the three TMBO/RGO samples; **(B)** FT-IR spectrum of NCBO/RGO-3; High-resolution XPS spectra of **(C)** Ni 2p, **(D)** Co 2p, **(E)** O 1s and **(F)** C 1s for NCBO/RGO-3.

The XRD patterns of the three TMBO/RGO samples are shown in Figure 4A. No obvious diffraction peaks are observed suggests the amorphous structure of CBO/RGO. The broad diffraction peaks of NBO/RGO at 34.2° and 61.3° can be attributed to the (202) and (303) crystal planes of Ni_3_(BO_3_)_2_ (PDF#26-1284), respectively, while the sharp diffraction peak at 44.7° corresponds to the (102) crystal plane of Ni_3_B (PDF#48-1223) (Chen et al., 2019a). In addition, NCBO/RGO-3 also shows a Ni_3_B diffraction peak at 44.7°, but its peak intensity is significantly weaker than that of NBO/RGO, indicating that the doping of Co elements significantly reduces the crystallinity of NCBO/RGO-3.

The FT-IR spectrum of NCBO/RGO-3 is shown in Figure 4B. The two peaks located at 3440 and 1631 cm^−1^ can be ascribed to the O-H stretching and bending vibrations of absorbed water within the material skeleton, respectively. It is found that two characteristic peaks appear at 1278 and 1423 cm^−1^ in NCBO/RGO-3, which indicates the existence of the B-O stretching vibration (Zhang et al., 2020). The infrared peak centered at 1112 cm^−1^ belongs to the vibration of SO_4_^2−^ residue in NCBO/RGO-3, while the infrared peaks located at 671 and 561 cm^−1^ are derived from the M-B (M = Co, Ni) bonds between the metal and boron atoms (Chen et al., 2017b).

The elemental compositions and binding energy of NCBO/RGO-3 were analyzed by XPS spectrum, as shown in Figures 4C–F. The spectrums of Ni 2p, Co 2p, and C 1s were computer fitted by the Gaussian fitting method. For the Ni 2p spectrum (Figure 4C), there are two satellite peaks at the higher binding energy of Ni 2p_3/2_ (856.7 eV) and Ni 2p_1/2_ main peaks (873.6 eV), respectively, indicating that the superficial nickel is mainly presented in the oxidation state (Ni^2+^ and Ni^3+^) (Zhang et al., 2019b; Wang et al., 2022). The above Ni^2+^ and Ni^3+^ are derived from nickel borate (labeled as Ni-B_i_) (Jiang et al., 2017; Chen et al., 2019b). Similarly, there are also two satellite peaks located approximately 5.5 eV above the major Co 2p_3/2_ (781.6 eV) and Co 2p_1/2_ main peaks (797.1 eV), respectively (Figure 4D), which are assigned to oxidized Co^2+^ and Co^3+^ species from cobalt borate (labeled as Co-B_i_) (Chen et al., 2015; Hu et al., 2019). The distinct peak at 192.4 eV can be attributed to the oxidized boron from the BO_3_^3-^ (Figure 2E), indicating that the oxidized boron species are in predominance on the shell of NCBO/RGO-3 (Chen et al., 2017a). Since oxygen is more electro-negative than boron, the superficial metal borides are easily oxidized upon exposure to air, and this phenomenon is also reported in many studies (Masa et al., 2016; Masa et al., 2017). It should be noted that XPS characterization is only sensitive to surface powders (a few nanometers), while it is difficult to analyze the structure of deeper powders. The previous HR-TEM image, XRD pattern, and FT-IR spectrum have demonstrated the existence of Ni-Co borides in NCBO/RGO-3. Therefore, NCBO/RGO-3 can be considered as NCB@NCB_i_/RGO composites with Ni-Co boride as the core layer and Ni-Co borate (NCB_i_) as the shell layer. These metal borides and borates can provide sufficient active sites for redox reactions, thus effectively improving the electrochemical properties of materials. In C 1s spectrum (Figure 4F), the characteristic peaks at 284.8, 286.4, and 288.9 eV correspond to C-C, C-O, and C=O bonds, respectively. The weak C-O and C=O bonds coupling with strong C-C bond indicate that most oxygen-containing functional groups on GO are removed after calcination, and hence the electrical conductivity of RGO can be greatly enhanced (Zhao et al., 2021a). Therefore, NCBO/RGO-3 is expected to achieve excellent electrochemical performance.

### 3.2 Electrochemical Measurements of Transition Metal Borides@Borates/RGO Samples

**FIGURE 5:**
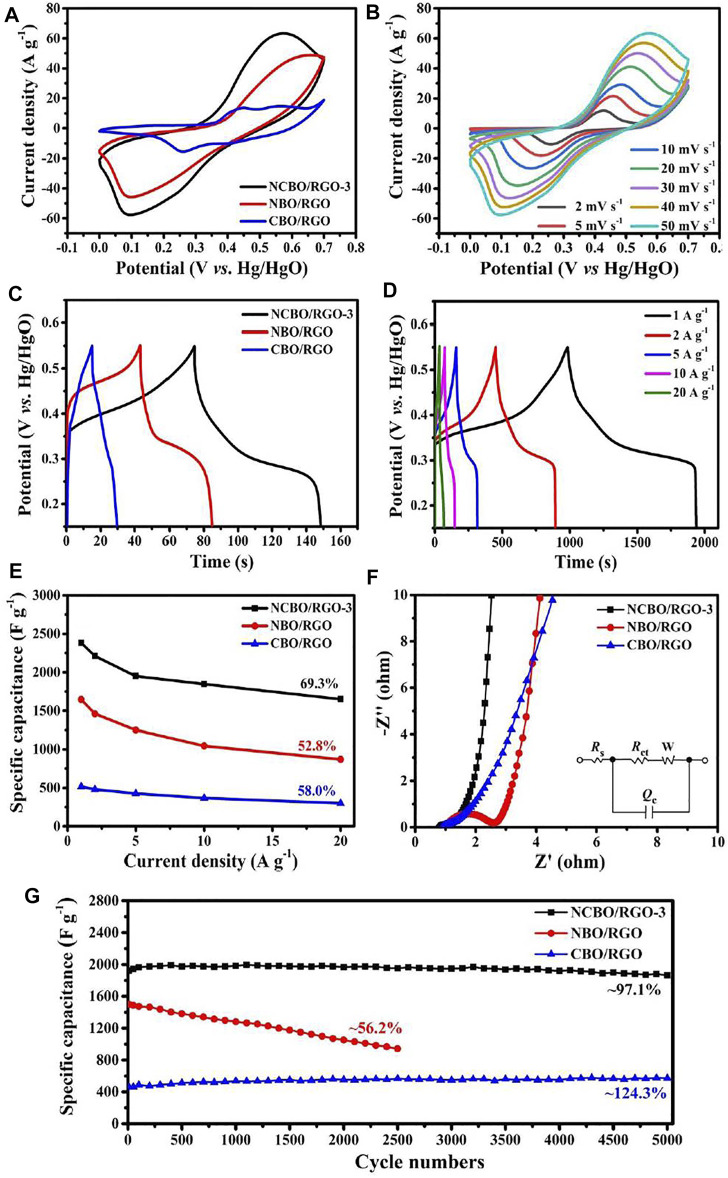
**(A)** CV curves of the three TMBO/RGO samples at 50 mV s^−1^; **(B)** CV curves of NCBO/RGO-3 at various scan rates; **(C)** GCD curves of the three TMBO/RGO samples at the current density of 10 A g^−1^; **(D)** GCD curves of NCBO/RGO-3 at different current densities; **(E)** Specific capacitances of the three TMBO/RGO samples at different current densities; **(F)** Nyquist plots of the three TMBO/RGO samples; **(G)** Cycling performances of the three TMBO/RGO samples at 5 A g^−1^.

Figure 5A shows the cyclic voltammetry (CV) curves of the three TMBO/RGO samples at the scan rate of 50 mV s^−1^. A pair of strong and well-defined redox peaks is visible for all three TMBO/RGO, which are mainly attributed to the M-B-related redox reactions. Among them, the CV curve of NCBO/RGO-3 with the largest surrounded area indicates its highest specific capacitance. When the scan rate is increased from 2 mV s^−1^ to 50 mV s^−1^, the shape of CV curves for the oxidation and reduction peaks of NCBO/RGO-3 is basically unchanged except for the increase of the respective peaks currents (Figure 5B), signifying that NCBO/RGO-3 electrode has excellent rate capability and electrochemical reversibility (Guo et al., 2020).

The galvanostatic charge-discharge (GCD) curves of the three TMBO/RGO samples at the current density of 10 A g^−1^ are shown in Figure 5C. It can be seen that the charging and discharging curves of NCBO/RGO-3 are nearly symmetrical and have the longest discharge time among the three TMBO/RGO samples, indicating a good electrochemical reversibility and the highest specific capacitance of NCBO/RGO-3. The GCD curve of NCBO/RGO-3 has an obvious plateau (Figure 5D), which again proves its pseudo-capacitance characteristics (Huo et al., 2019). Figure 5E shows the specific capacitances of the three TMBO/RGO samples at different current densities. At the current density of 1 A g^−1^, the specific capacitances of NCBO/RGO-3, NBO/RGO, and CBO/RGO are 2383, 1647, and 517.5 F g^−1^, respectively. When the current density increases to 20 A g^−1^, NCBO/RGO-3 still maintains 69.3% of specific capacitance and is much better than NBO/RGO and CBO/RGO in terms of specific capacitance and rate capability. In addition, the supercapacitive performances of the as-prepared NCBO/RGO composites are greatly influenced by their Ni/Co molar ratios. It is found that NCBO/RGO-0.5, whose Ni/Co molar ratio is 0.5, displays a specific capacitance of 1415.5 F g^−1^ that much higher than CBO/RGO (Supplementary Figures S4A–C). With an increase of Ni/Co ratio, the specific capacitances of NCBO/RGO composites gradually increase first and then decrease, and the highest specific capacitance is obtained with NCBO/RGO-3. Additionally, the rate capability of NCBO/RGO-3 is much better than those of NCBO/RGO composites with other Ni/Co ratios when the current density is increased to 20 A g^−1^ (Supplementary Figure S4C). Therefore, the optimal Ni/Co molar ratio is determined to be 3.

The electrochemical impedance spectroscopies (EIS) of the three TMBO/RGO samples in the frequency range of 0.001 Hz–100 kHz are shown in Figure 5F. The intersection of the semicircular ring on the Z′ axis in the high-frequency region represents the solution resistance *R*_s_ of the test system, while the diameter of semicircular ring determines the electron transfer resistance *R*_ct_, and the angle of the slope in the low-frequency region is related to the Warburg impedance. In terms of *R*_s_, NCBO/RGO-3, NBO/RGO, and CBO/RGO are close because all three electrodes are evaluated in the same electrolyte. The electron transfer resistances (*R*_ct_) of NCBO/RGO composites are much smaller than that of NBO/RGO (*R*_ct_ = 1.49 Ω), which suggests that the doping of Co elements can significantly promote charge transfer (Figure 5F and Supplementary Figure S4D). In addition, NCBO/RGO-3 has a nearly vertical Warburg angle as compared to CBO/RGO, indicating faster electrolyte ion diffusion within the NCBO/RGO-3 electrode (Zhang et al., 2020). It also has been suggested that the M_3_(BO_3_)_2_ (M = Ni, Co) shell layer promotes the absorption of electrolyte OH^−^ on the electrode surface (Chen et al., 2019b), and the abundant mesopores inside the NCBO/RGO-3 can facilitate the transport and infiltration of electrolyte ions within the whole electrode material. Therefore, NCBO/RGO-3 exhibits excellent specific capacitance and rate capability.

Cycling stability is an important index to evaluate the performance of supercapacitors. The cycling stability of the three TMBO/RGO samples at 5 A g^−1^ is shown in Figure 5G. For NBO/RGO, significant capacitance degradation is observed with only 56.2% of the initial capacitance remaining after 2500 charge/discharge cycles. The reason is that the loading particles of NBO/RGO are relatively large and poorly conductive, and the electrode material is easily pulverized and then delaminated from the current collector during the fast charge/discharge processes. CBO/RGO possesses low capacitance but good stability with a gradual increment in specific capacitance during 5000 charge/discharge cycles, mainly because the electrolyte ions slowly infiltrates into the internal pores to fully activate the electrode during cycling (Zhang et al., 2019b). In contrast, NCBO/RGO-3 still maintains 97.1% of the initial capacitance after 5000 charge/discharge cycles, which displays a gratifying capacitance, an excellent rate capability and a superior cycling stability that outperform most TMBOs in literatures (Supplementary Table S1). The excellent supercapacitive properties of NCBO/RGO-3 synthesized by TS-IJMR are mainly due to the following reasons: 1) NBO, CBO, and RGO in NCBO/RGO-3 produce a good synergistic effect, and the doping of Co enhances the electrical conductivity of the material; 2) The 2D conductive network of RGO facilitates the electron transfer throughout the electrode; 3) The RGO sheet not only suppresses the material deformation and structural collapse of NCBO-3 during the fast charge/discharge processes but also enables the uniformly dispersed NCBO-3 particles to achieve a high electroactive surface area; 4) The M_3_(BO_3_)_2_ shell layer of NBCO/RGO-3 contributes to the adsorption of electrolyte ions, and abundant mesopores can facilitate the transport and infiltration of electrolyte within the whole electrode.

### 3.3 Comparisons of Nickel-Cobalt Boride@Borate/RGO Generated by Different Precipitation Methods

It can be seen from Supplementary Figure S5 that the supercapacitive performance of NCBO/RGO-3 synthesized in TS-IJMR is much better than that of NCBO/RGO-S synthesized in STR. The supercapacitive performance of electrode material is mainly determined by its morphology, specific surface area, pore size distribution, etc. (Simon and Gogotsi, 2008), which can be further influenced by the different mixing methods of the two reactors. As mentioned above, during the precipitation of nanomaterials in STR, the reactant solutions are added dropwise into the reactor at a certain rate, and then the concentration and pH in STR are homogenized by constant agitation. The feeding rate should not be too fast due to the poor micromixing efficiency of STR (Bhattacharya and Kresta, 2004), hence it usually takes dozens of minutes or even hours to complete the whole precipitation process. However, even if the feeding rate is strictly controlled to achieve a nearly uniform supersaturation in STR by vigorous agitation, the local concentration, pH, and supersaturation are neither spatially nor temporally constant when a drop of reactant solution is added into the STR, in which the previously generated precipitates and dissolved ions have already coexisted. In addition, the newly added metal ions will adsorb on the surface of the previously generated precipitates to form bigger aggregates (Cheng et al., 2009; Alexandrov, 2014). As a consequence, the NCBO/RGO-S synthesized in STR is severely agglomerated (Figure 2F), with a BET specific surface area of only 36.73 m^2^ g^−1^ (Supplementary Figure S6). This makes substantial particle surfaces cannot be infiltrated by the electrolyte, hence reducing the supercapcitive performance of NCBO/RGO-S. In contrast, TS-IJMR has good micromixing efficiency, and Ni^2+^/Co^2+^ solution can quickly achieve homogeneous mixing with GO sheets at the first micro T-junction. Then, the mixture re-impacts with KBH_4_ solution at high velocities to obtain uniformly loaded NCBO particles on GO flakes (Guo et al., 2017). In addition, TS-IJMR provides a more homogeneous and stable environment for both particle nucleation and crystal growth on account of its better process control and enhanced micromixing efficiency. Therefore, the as-prepared NCBO/RGO-3 possesses a large BET specific surface area (62.37 m^2^/g), quantities of suitable mesopores (2–5 nm) and fast ion transport to participate in redox reactions. As a consequence, the supercapacitive performance of NCBO/RGO-3 is much superior to that of NCBO/RGO-S synthesized in STR.

### 3.4 Electrochemical Measurements of Nickel-Cobalt Boride@Borate/RGO//AC Asymmetric Supercapacitor

**FIGURE 6:**
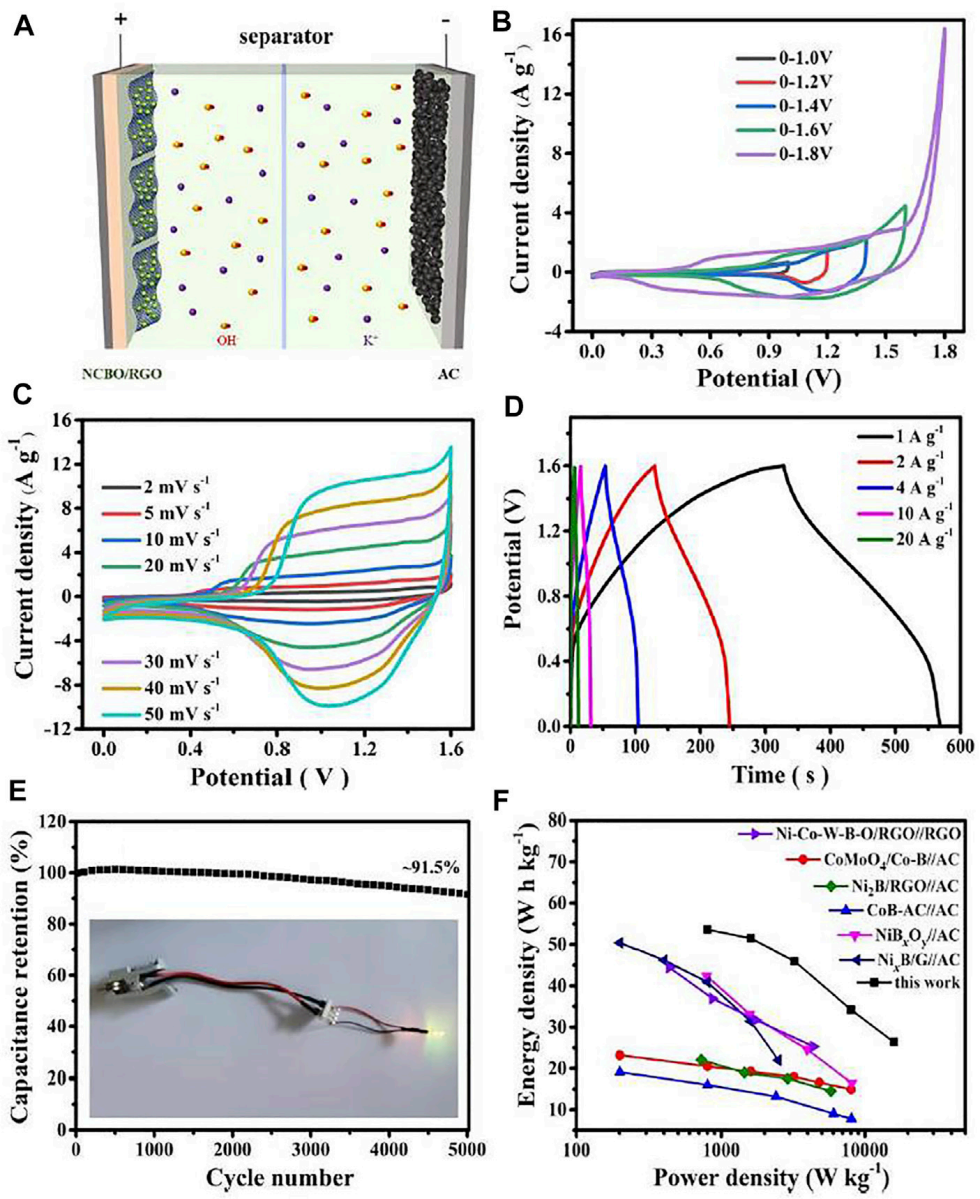
**(A)** The electrochemical character of NCBO/RGO//AC asymmetric supercapacitor; **(B)** CV curves of NCBO/RGO//AC device test at different voltage windows (20 mV s^−1^); **(C)** CV curves of NCBO/RGO//AC device at various scan rates; **(D)** GCD curves of NCBO/RGO//AC device at various current densities; **(E)** Long-term cycling test of NCBO/RGO//AC device at 2 A g^−1^ (inset: photograph of a yellow LED lighted with two tandem NCBO/RGO//AC asymmetric supercapacitors); **(F)** Ragone plot of NCBO/RGO//AC device compared with other literatures.

Although NCBO/RGO-3 displays a high specific capacitance, the operating voltage of 0.4 V still hampers its practical applications for energy storage. In order to broaden the operating voltage of the NCBO/RGO-3, the button-type asymmetric supercapacitor NCBO/RGO//AC is assembled with the NCBO/RGO-3 electrode and AC electrode as the positive electrode and negative electrode, respectively (Figure 6A). The CV curves of NCBO/RGO//AC device at different voltage windows are shown in Figure 6B. When the voltage window is increased to 1.8 V, an obvious hump is observed within the potential of 1.6–1.8 V, suggesting that OER takes place in the positive electrode. Therefore, the optimal voltage window for NCBO/RGO//AC device is determined to be 0–1.6 V. The CV shapes does not change significantly as the scan rates increases (Figure 6C), which signifies the fast charge/discharge responses of NCBO/RGO//AC device. In addition, the nearly symmetrical charge-discharge curves of NCBO/RGO//AC further validates its good charge/discharge reversibility and high Coulombic efficiency (Figure 6D). The specific capacitances of NCBO/RGO//AC are 150.8, 144.8, 129.2, 96.1, and 74.3 F g^−1^ at the current densities of 1, 2, 4, 10, and 20 A g^−1^, respectively, coupling with 91.5% capacitance retention after 5000 charge-discharge cycles at 2 A g^−1^ (Figure 6E). Therefore, the assembled NCBO/RGO//AC device displays a good rate capability and cycling stability.

Energy density and power density are two important indicators of supercapacitor performance. Figure 6F demonstrates that the NCBO/RGO//AC device provides a maximum energy density of 53.3 W h kg^−1^ at a power density of 800 W kg^−1^. Even at a high power density of 16,000 W kg^−1^, it can still achieve an energy density of 26.4 W h kg^−1^, which is higher than that previously reported TMBO-based asymmetric supercapacitors, such as CoMoO_4_/Co-B//AC (Hou et al., 2020), CoB-AC//AC (Hou et al., 2019), NiB_*x*_O_*y*_//AC (Qin et al., 2018), Ni_2_B/RGO//AC (Cao et al., 2017), Ni_*x*_B/G//AC (Chen et al., 2019b), Ni-Co-W-B-O/RGO//RGO (Xiang et al., 2017). Finally, a yellow LED was lighted for several minutes with two tandem NCBO/RGO//AC devices. Therefore, the NCBO/RGO-3 electrode materials we synthesized have promising prospects for application in the field of energy storage.

### 3.5 Oxygen Evolution Reaction Measurement of Nickel-Cobalt Boride@Borate/RGO

**FIGURE 7:**
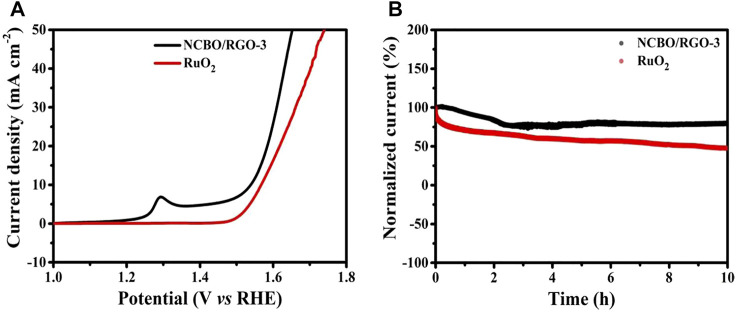
**(A)** LSV curves of NCBO/RGO-3 and commercial RuO_2_ catalyst; **(B)** The stability tests of NCBO/RGO-3 and commercial RuO_2_ catalyst at the current density of 10 mA cm^−2^.

In order to explore the potential applications of NCBO/RGO-3 in electrocatalysis, the OER performance of NCBO/RGO-3 was evaluated. The LSV curve of NCBO/RGO-3 is shown in Figure 7A. At a current density of 10 mA cm^−2^, NCBO/RGO-3 can obtain an overpotential as low as 309 mV, which is lower compared to the RuO_2_ reference catalyst (338 mV), indicating that NCBO/RGO-3 has better catalytic performance than RuO_2_.

Stability is also a key parameter to evaluate the performance of OER electrocatalysts. As shown in Figure 7B, the NCBO/RGO-3 catalyst only slightly decays after 10 h of stable cycling at a constant potential of 1.54 V, with the current remaining above 80%. In contrast, commercial RuO_2_ catalysts show a sharp current decay at 10 h. Therefore, NCBO/RGO-3 catalyst has good stability in OER. The NCBO-3 grown on the RGO sheet facilitates the exposure of more active sites on the electrocatalyst surface, thus accelerating the OER reaction at the solid-liquid interface. Moreover, the synergistic effect of the components in NCBO/RGO-3 also plays a facilitating role. Therefore, NCBO/RGO-3 represents an OER electrocatalyst with potential applications.

## 4 Conclusion

A novel TS-IJMR is constructed for the controllable and scale-up synthesis of NCBO/RGO-3 as both the supercapacitor electrode material and OER electrocatalyst. Benefiting from the precise process control and high micromixing efficiency of TS-IJMR, the NCBO/RGO-3 nanosheet contains a large BET specific surface area and abundant suitable mesopores (2–5 nm), and have an intimate electrical connection between the NCBO-3 particles and RGO flakes. Therefore, fast ion diffusions, facile electron transfer, quantities of superficial electroactive sites, and strong synergistic effects between NCBO-3 and RGO sheets make great contributions to the reversible redox reactions, which finally delivers a high specific capacitance of 2383 F g^−1^ at a current density of 1 A g^−1^, as well as good rate capability and cycling stability. The supercapacitive performance of NCBO/RGO-3 is much better than that of NBO/RGO and CBO/RGO synthesized in TS-IJMR and NCBO/RGO-S synthesized in STR. The asymmetric supercapacitor NCBO/RGO//AC, obtained by assembling NCBO/RGO-3 with AC, has an energy density of 53.3 W h kg^−1^ and capacitance retention of 91.8% after 5000 fast charge/discharge cycles. Finally, NCBO/RGO-3 is used as an OER electrocatalyst to obtain overpotentials as low as 309 mV at a current density of 10 mA cm^−2^. Its current remains above 80% after 10 h of stable cycling at a constant voltage of 1.54 V. In conclusion, NCBO/RGO-3 synthesized by TS-IJMR on a large scale is a promising electrode material for energy storage and conversion.

## Data Availability

The original contributions presented in the study are included in the article/Supplementary Material, further inquiries can be directed to the corresponding authors.
