# Supplementary material for: Controllable and Scale-Up Synthesis of Nickel-Cobalt Boride@Borate/RGO Nanoflakes via Reactive Impingement Mixing: A High-Performance Supercapacitor Electrode and Electrocatalyst
Source: Front Chem. 2022 Apr 12;10:874675. doi: 10.3389/fchem.2022.874675 (PMC9039022; doi:10.3389/fchem.2022.874675)
Supplement: Supplementary file 1 [file DataSheet1.docx]

**Controllable and Scale-up Synthesis of Nickel-Cobalt Boride@Borate/RGO Nanoflakes via Reactive Impingement Mixing: A High-performance Supercapacitor Electrode and Electrocatalyst**

Yudan Qian,^1†^ Yechao Wu,^1†^ Fan Gu,^1†^ Zhiming Zhou,^1^ Zaimei Huang,^1^ Xinyue Tang,^1^ Shuang Pan,^1^ Shangcong Zhang,^2,3^* Shinan Chen,^4^ Qingcheng Zhang,^1^* Yihuang Chen,^1^* Shun Wang^1^

*^1^College of Chemistry and Materials Engineering, Wenzhou University, Zhejiang 325035, China*

*^2^* *Low Voltage Apparatus Technology Research Center of Zhejiang, Wenzhou University, Zhejiang, 325035, China*

*^3^Technology Institute of Wenzhou University in Yueqing, 325600, Zhejiang, China*

*^4^Zhejiang Zheneng Wenzhou Electric Power Generation Co., LTD.,325602, Zhejiang, China*

^*^Corresponding author. E-mail: [zsc@wzu.edu.cn](mailto:zsc@wzu.edu.cn) (Shangcong Zhang)

[zhangqc@wzu.edu.cn](mailto:zhangqc@wzu.edu.cn) (Qingcheng Zhang)

[yhchen@wzu.edu.cn](mailto:yhchen@wzu.edu.cn) (Yihuang Chen)

**^†^** *These authors have contributed equally to this work*


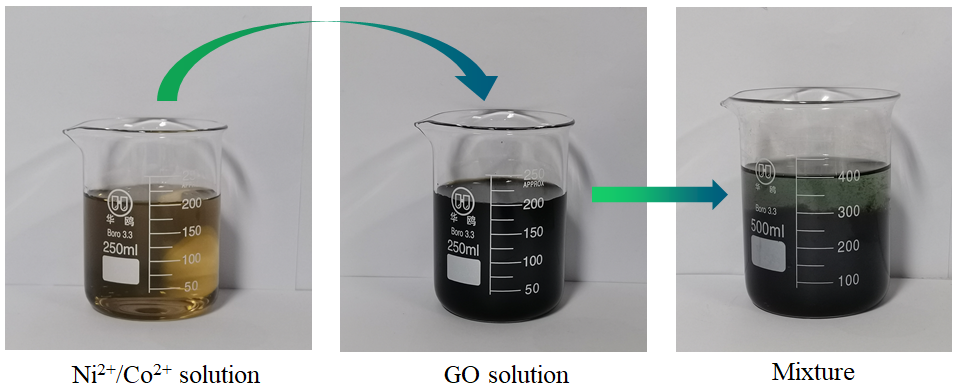


Figure S1. The serious coagulation of Ni^2+^/Co^2+^/GO mixture.


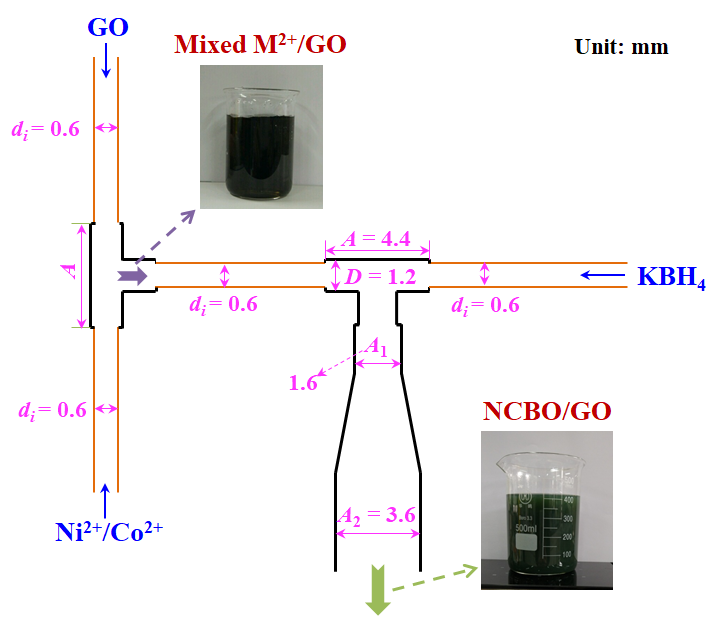


Figure S2. Geometric structure of TS-IJMR


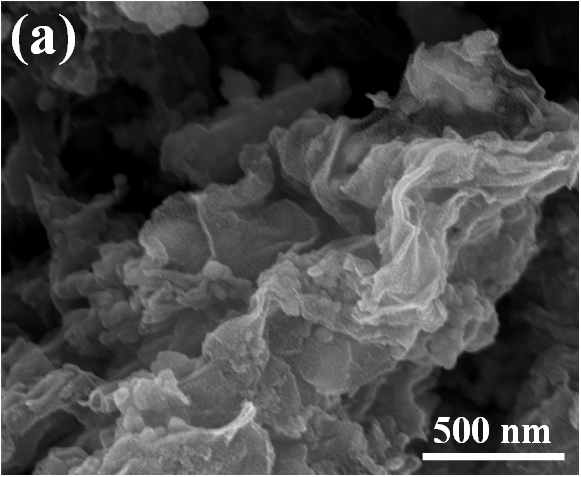

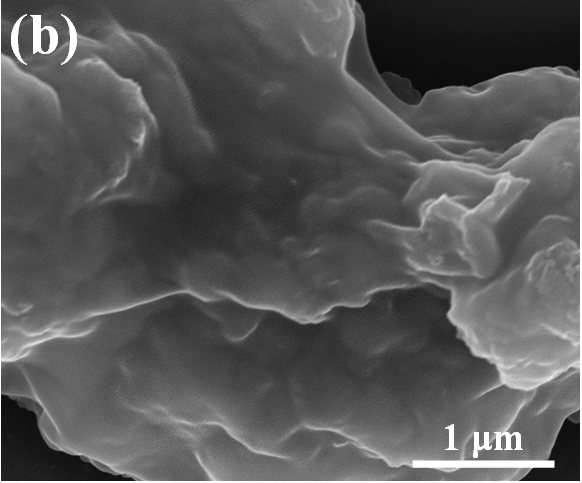

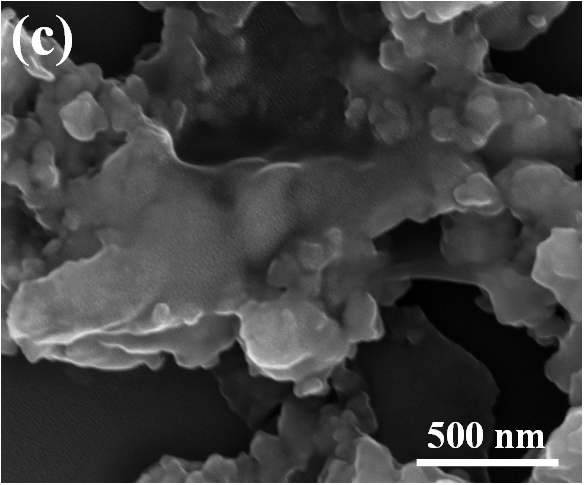


Figure S3. (a)-(c) SEM images of NBO/RGO, CBO/RGO and NCBO/RGO-3, respectively


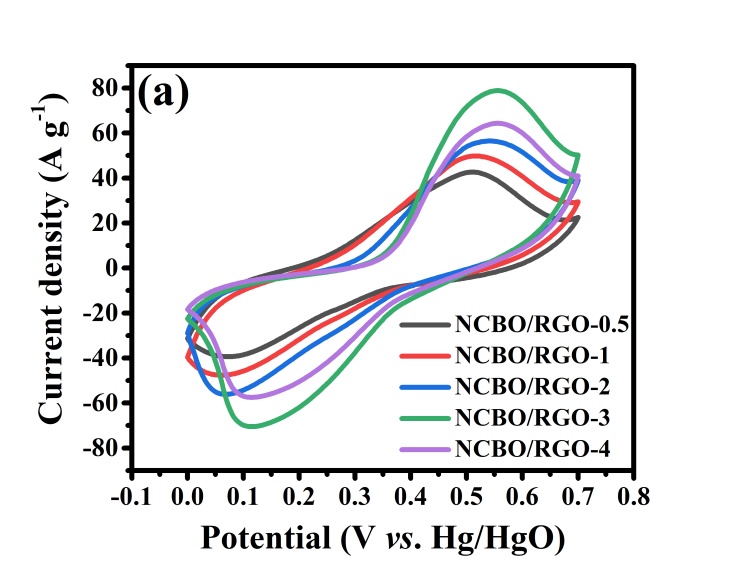

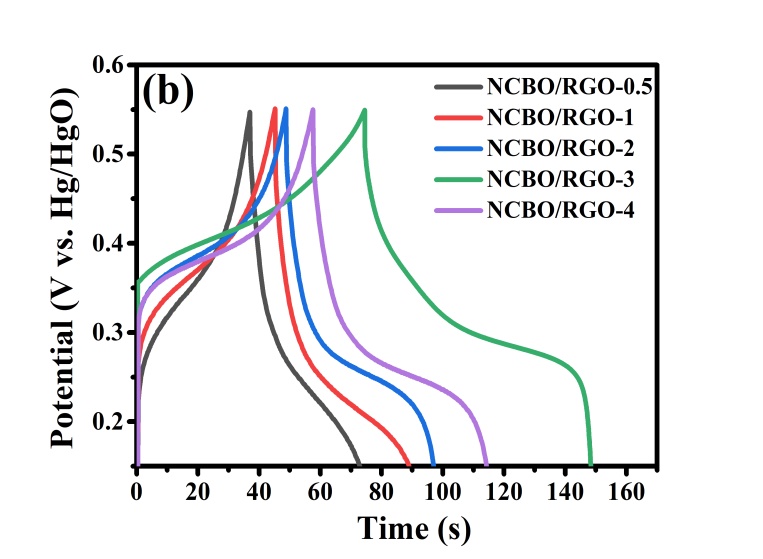


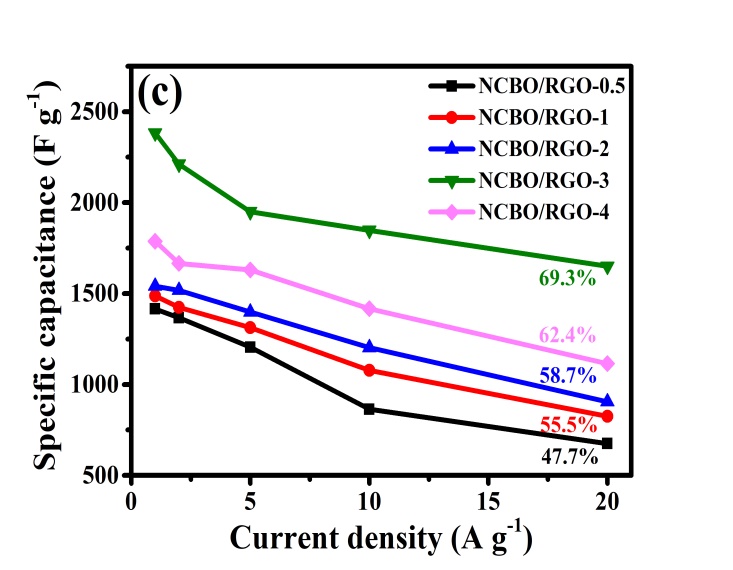

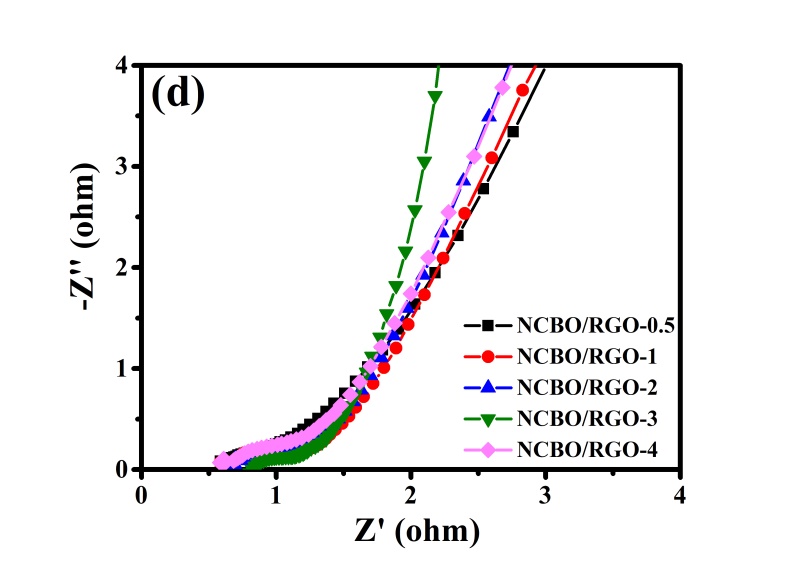


Figure S4. (a) CV curves of NCBO/RGO composites with various Ni/Co ratios (50 mV s^-1^); (b) GCD curves of NCBO/RGO composites with various Ni/Co ratios (10 A g^-1^); (c) Rate capability of NCBO/RGO composites with various Ni/Co ratios; (d) EIS spectra of NCBO/RGO composites with various Ni/Co ratios.


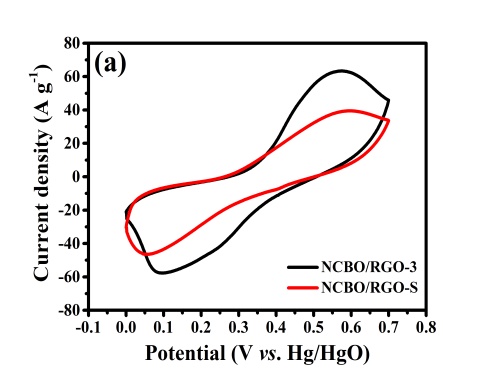

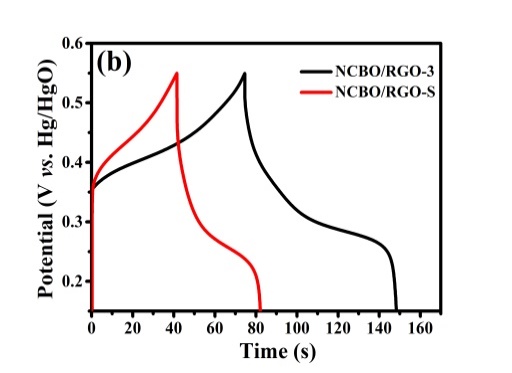

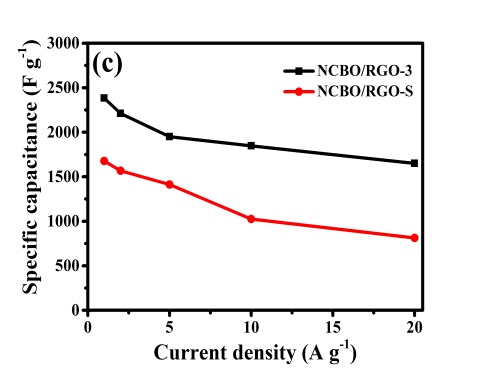


Figure S5. (a) CV curves of NCBO/RGO and NCBO/RGO-S at 50 mV s^-1^; (b) GCD curves of NCBO/RGO and NCBO/RGO-S at the current density of 10 A g^-1^; (c) Specific capacitances of NCBO/RGO and NCBO/RGO-S at different current densities.


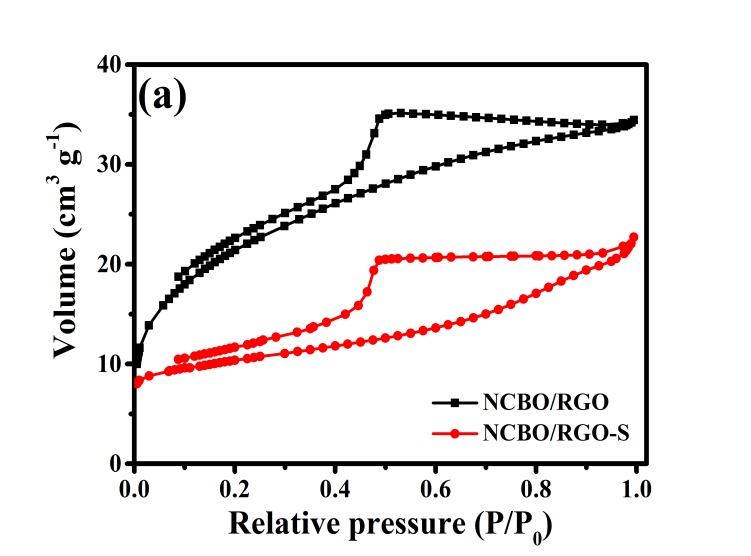

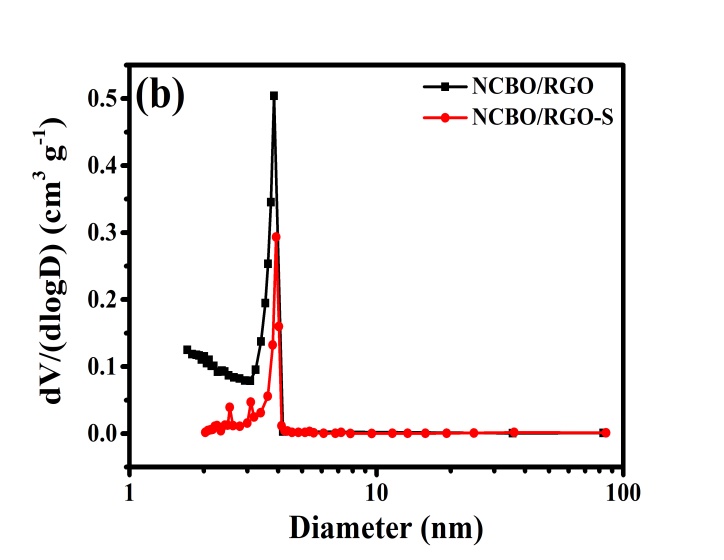


Figure S6. (a) N_2_ adsorption/desorption isotherms; (b) Pore size distributions of NCBO/RGO and NCBO/RGO-S.

Table S1. Comparison of the supercapacitive performances of metal borides or borates.

| Materials | Specific capacitance  (F g^-1^) | Rate capability | Cycle stability  (cycle number) | References |
| --- | --- | --- | --- | --- |
| Ni-B | 2230 (1 A g^-1^) | 44.2% (1→20 A g^-1^) | 97.9% (2000) | (Li et al., 2017) |
| Co-Ni-B/RGO/fabric | 276.9 (0.25 mA cm^-2^) | 22.8% (0.25→1 mA cm^-2^) | 85.0% (3000) | (Wang et al., 2020) |
| Ni*_x_*B/G | 1822 (1 A g^-1^) | 64.7% (1→20 A g^-1^) | 96.0% (2000) | (Chen et al., 2019) |
| Co–Ni–W–B–O/rGO | 1189.1 (1 A g^-1^) | 56.2% (1→15 A g^-1^) | 80.7% (10000) | (Xiang et al., 2017) |
| Ni-Co-B-O | 1139 (1 A g^-1^) | 58.7% (1→25 A g^-1^) | 83.5% (2000) | (Qiu et al., 2020) |
| Ni-B-O | 855 (0.2 A g^-1^) | 55.9% (0.2→5 A g^-1^) | ------ | (Qin et al., 2018) |
| Ni_2_B/RGO | 1073.4 (1 A g^-1^) | 78.3% (1→6 A g^-1^) | 66.4% (2500) | (Cao et al., 2017) |
| CoB@Ni(OH)_2_ | 1504.4 (0.5 A g^-1^) | 49.6% (0.5→6 A g^-1^) | 85.0% (2500) | (Wang et al., 2017) |
| Ni_3_V_2_O_8_@Co-B | 1789 (0.5 A g^-1^) | 62.0% (0.5→10 A g^-1^) | ------ | (Hou et al., 2021) |
| Co-Fe-B | 981 (1 A g^-1^) | 73.8% (1→20 A g^-1^) | 73.0% (3000) | (Meng et al., 2019) |
| CoB-AC | 412 (0.5 A g^-1^) | 75.2% (0.5→10 A g^-1^) | 66.7% (10000) | (Hou et al., 2019) |
| Ni-Co-B-O/RGO | 2383 (1 A g^-1^) | 69.3% (1→20 A g^-1^) | 97.1% (5000) | This work |

**References**

Cao, X., Wang, X., Cui, L., Jiang, D., Zheng, Y., Liu, J., (2017). Strongly coupled nickel boride/graphene hybrid as a novel electrode material for supercapacitors. *Chem. Eng. J.* 327, 1085-1092. doi: 10.1016/j.cej.2017.07.010

Chen, Y., Zhou, T., Li, L., Pang, W.K., He, X., Liu, Y.-N., et al., (2019). Interfacial engineering of nickel boride/metaborate and its effect on high energy density asymmetric supercapacitors. *ACS Nano* 13, 9376-9385. doi: 10.1021/acsnano.9b04005

Hou, J.-F., Gao, J.-F., Kong, L.-B., (2019). Liquid phase reduction synthesis of a cobalt boride–activated carbon composite with improved specific capacitance and retention rate as a new positive electrode material for supercapacitors. *New J. Chem.* 43, 14475-14484. doi: 10.1039/C9NJ02830G

Hou, J.-F., Gao, J.-F., Kong, L.-B., (2021). A crystalline nickel vanadium oxide@amorphous cobalt boride nanocomposites with enhanced specific capacity for hybrid supercapacitors. *Electrochim. Acta* 377, 138086. doi: 10.1016/j.electacta.2021.138086

Li, W., Wang, S., Wu, M., Wang, X., Long, Y., Lou, X., (2017). Direct aqueous solution synthesis of an ultra-fine amorphous nickel–boron alloy with superior pseudocapacitive performance for advanced asymmetric supercapacitors. *New J. Chem.* 41, 7302-7311. doi: 10.1039/C7NJ00222J

Meng, Q., Xu, W., Zhu, S., Liang, Y., Cui, Z., Yang, X., et al., (2019). Low-cost fabrication of amorphous cobalt-iron-boron nanosheets for high-performance asymmetric supercapacitors. *Electrochim. Acta* 296, 198-205. doi: 10.1016/j.electacta.2018.11.067

Qin, W., Liu, Y., Liu, X., Yang, G., (2018). Facile and scalable production of amorphous nickel borate for high performance hybrid supercapacitors. *J. Mater. Chem. A* 6, 19689-19695. doi: 10.1039/C8TA07385F

Qiu, P., Kong, C., Ju, M., Zhang, J., Chen, X., (2020). Tuning oxidation degrees of low-crystallinity porous Ni–Co–B–O/C nanocomposites for high-performance hybrid supercapacitors. *Energy Fuels* 34, 16893-16902. doi: 10.1021/acs.energyfuels.0c03518

Wang, H., Yan, J., Wang, R., Li, S., Brett, D.J.L., Key, J., et al., (2017). Toward high practical capacitance of Ni(OH)_2_ using highly conductive CoB nanochain supports. *J. Mater. Chem. A* 5, 92-96. doi: 10.1039/C6TA08796E

Wang, W., Zhang, J., Li, T., Wang, S., (2020). Facile construction of a flexible and wearable electrode based on the hierarchical structure of RGO-coated cotton fabric with amorphous Co–Ni–B alloy. *RSC Adv.* 10, 43109-43116. doi: 10.1039/D0RA06988D

Xiang, C., Wang, Q., Zou, Y., Huang, P., Chu, H., Qiu, S., et al., (2017). Simple synthesis of graphene-doped flower-like cobalt–nickel–tungsten–boron oxides with self-oxidation for high-performance supercapacitors. *J. Mater. Chem. A* 5, 9907-9916. doi: 10.1 039/c7ta00234c
